# Characterization of the gastrointestinal microbiome of the Syrian hamster (*Mesocricetus auratus*) and comparison to data from mice

**DOI:** 10.1002/2211-5463.13869

**Published:** 2024-08-04

**Authors:** Linda F. Böswald, Bastian Popper, Dana Matzek, Klaus Neuhaus, Jasmin Wenderlein

**Affiliations:** ^1^ Core Facility Animal Models, Biomedical Center, Medical Faculty LMU Munich Planegg‐Martinsried Germany; ^2^ Core Facility Microbiome, ZIEL Institute for Food & Health Technical University of Munich Freising Germany; ^3^ Chair for Bacteriology and Mycology, Department of Veterinary Sciences, Faculty of Veterinary Medicine, Institute for Infectious Diseases and Zoonoses LMU Munich Oberschleißheim Germany; ^4^ Department for Biological Safety Federal Institute for Risk Assessment Berlin Germany

**Keywords:** 16S rRNA gene amplicon sequencing, gastrointestinal microbiome, microbiome, mouse, rodent microbiome, Syrian hamster

## Abstract

Syrian hamsters (*Mesocricetus auratus*) have been increasingly used as rodent models in recent years, especially for SARS‐CoV‐2 since the pandemic. However, the physiology of this animal model is not yet well‐understood, even less when considering the digestive tract. Generally, the gastrointestinal microbiome influences the immune system, drug metabolism, and vaccination efficacy. However, a detailed understanding of the gastrointestinal microbiome of hamsters is missing. Therefore, we analyzed 10 healthy 11‐week‐old RjHan:AURA hamsters fed a pelleted standard diet. Their gastrointestinal content was sampled (i.e., forestomach, glandular stomach, ileum, cecum, and colon) and analyzed using 16S rRNA gene amplicon sequencing. Results displayed a distinct difference in the bacterial community before and after the cecum, possibly due to the available nutrients and digestive functions. Next, we compared hamsters with the literature data of young‐adult C57BL/6J mice, another important animal model. We sampled the same gastrointestinal regions and analyzed the differences in the microbiome between both rodents. Surprisingly, we found strong differences in their specific gastrointestinal bacterial communities. For instance, *Lactobacillaceae* were more abundant in hamsters' forestomach and ileum, while *Muribaculaceae* dominated in the mouse forestomach and ileum. Similarly, in mouse cecum and colon, *Muribaculaceae* were dominant, while in hamsters, *Lachnospiraceae* and *Erysipelotrichaceae* dominated the bacterial community. Molecular strains of *Muribaculaceae* in both rodent species displayed some species specificity. This comparison allows a better understanding of the suitability of the Syrian hamster as an animal model, especially regarding its comparability to other rodent models. Thereby, this work contributes to the characterization of the hamster model and allows better experimental planning.

## Graphical abstract

To gain insight into the Syrian hamsters' (RjHan:AURA) microbiome, different gastrointestinal sections were analyzed using 16S rRNA gene amplicon sequencing. Distinct differences in bacterial communities before and after the cecum were found. Comparison to data from C57BL/6J mice displayed strong differences. This comparison allows a better understanding of the suitability and comparability of the Syrian hamster as an animal model.

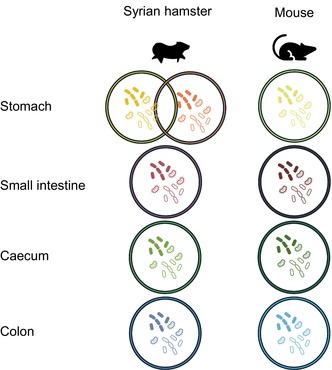

**Abbreviations**
AG: anterior gastrointestinal compartments
BC: bacterial community
CAE: content of cecum
CH‐Index: Calinski–Harabasz Index
COL: content of colon
FS: content of nonglandular forestomach
GS: content of glandular stomach
H2‐H11: hamster H2–hamster 11
MDS: multidimensional scaling plot
OTU: operational taxonomic units
PG: posterior gastrointestinal compartments
SCFA: short‐chained fatty acids
SIT: content of small intestinal tract
zOTU: zero‐radius operational taxonomic units

Currently, a gap in knowledge regarding the degree of suitability and the limitations of data interpretation originating from laboratory rodent trials is observed [1]. In laboratory mice, this knowledge gap has been recognized, and various publications describe the differences between mouse and human genetics [1, 2], immunology [3, 4], and discuss the question of suitability for mouse models in certain research areas. Differences in anatomy and physiology between mice and men cause issues for experimental reproducibility and standardization. The influence of the gut microbiome is scrutinized [5, 6]. Considering the differences in nutrition and gastrointestinal anatomy, it is not surprising that mouse models hardly reproduce the human intestinal microbiome [1]. This is aggravated by several issues: The murine gastrointestinal physiology is not yet completely understood [7]. Several common bacterial species in the mouse intestine are still lacking a detailed description [8, 9, 10]. In addition, the influence of environmental and dietary factors on the gastrointestinal microbiome of mice are still mostly unknown (e.g., breeder, housing conditions, diet composition, and processing) [11, 12]. While researchers try to close this knowledge gap for mice, the Syrian hamster is less well‐understood, and research on this species´ digestive physiology and microbiome is warranted. Syrian hamsters (*Mesocricetus auratus*) are being used as animal models for various bacterial, viral, and protozoal diseases, as well as in cancer research [13, 14, 15, 16, 17]. In the past years, this species has increasingly been used as an animal model due to a similar pathophysiology of the human COVID‐19 disease. Although, when using model organisms, it is important to have a clear understanding of their anatomy, physiology, and genetics to plan animal experiments, and to draw the correct conclusions from the results, there still is a knowledge gap. This knowledge is important to ensure the 3R (Replacement, Reduction, Refinement) and reproducibility [18] as well as the internal and external validity of animal experiments [19].

Mice and Syrian hamsters consume a mostly granivorous diet, while the human diet can be characterized as mostly omnivorous, with the dominance of plant‐ or meat‐based food depending on sociocultural factors [6]. Accordingly, the gastrointestinal tract of mice and hamsters shows several adaptations to the natural diet [20]. Both rodents are classified as hindgut fermenters [20, 21]. Mice have a rather simple stomach, with a small nonglandular part that is not distinctly separated from the glandular part, and an enlarged cecum as a fermentation chamber [6, 22]. In contrast, in Syrian hamsters, both the forestomach and the cecum are enlarged [23]. These adaptations to the natural diet [20] might be connected with the gastrointestinal microbiome as bacterial fermentation of the undigestible fraction of nutrients takes place in the cecum [20, 21, 22].

Starch is the major source of energy in grain‐based diets, and its digestion follows an overall similar process with species‐specific peculiarities. Depending on the degree of starch gelatinization, starch is digested by pancreatic amylase in the small intestine or fermented microbially in the cecum of the hindgut fermenters [7, 12]. Recent results on the pancreatic amylase activity of mice were in the expected range for such a granivorous species [24]. The murine colon is rather short and simple [21]. In hamsters, the stomach is distinctly compartmentalized and composed of a nonglandular forestomach and a glandular stomach proportion [22, 25]. These parts are separated by a sphincter muscle [26]. The digestive physiology of hamsters, especially of the forestomach, is not yet well‐understood. Nevertheless, evidence for bacterial presence and fermentation activity [27, 28] supports the hypothesis of the hamster forestomach being a fermentation chamber. While the digestion of fiber in this compartment has previously been negated [23, 29], staining with Lugol's iodine [24] supports starch fermentation already in the forestomach. Figure 1 gives a simplified, schematic overview of the anatomic structures with their main functions in Syrian hamsters and mice.

**Fig. 1:**
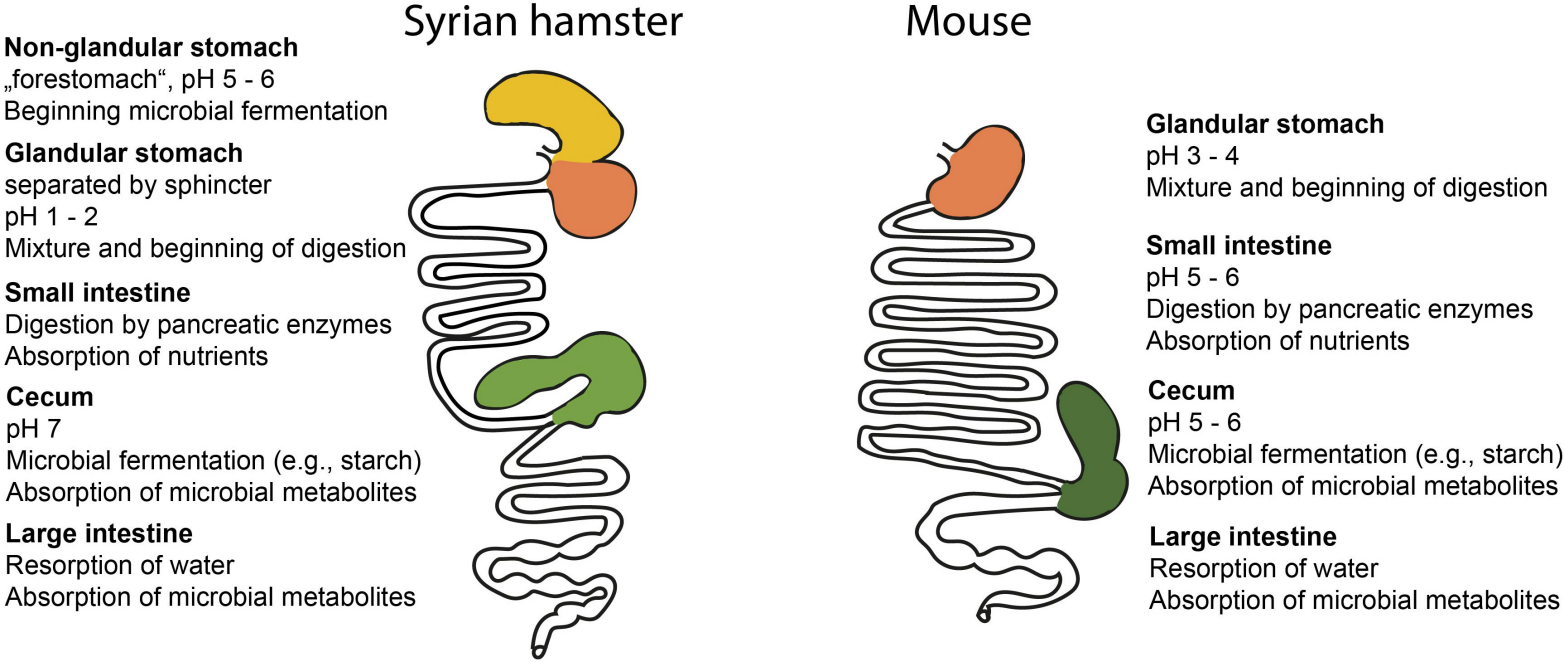
Schematic overview of the gastrointestinal tract of Syrian hamster and mouse with a comparative description of the main functions of each compartment (figure drawn by L. Böswald, adapted with permission from Stevens and Hume [20] with literature information added [6, 7, 12, 21, 22, 23, 24, 26, 66, 67, 68]).

The human gastrointestinal tract differs considerably from that of mice and hamsters. As said, humans are omnivorous and adapted to rather highly digestible diets. They have a simple glandular stomach and rely mostly on prececal digestion and absorption of nutrients. Bacterial fermentation of dietary substrates takes place mostly in the large intestine and to a lesser extent compared with rodents. The cecum of humans is rudimentary and does not contribute vitally to the digestive process. The difference in gastrointestinal anatomy between mice and men has been reviewed in great detail by Nguyen *et al*. [30].

As mentioned above, mice and hamsters, as hindgut fermenters, rely on bacterial fermentation to efficiently utilize their diets. This is conducted by members of the microbiome, which is defined as the totality of microorganisms (i.e., archaea, bacteria, fungi, protozoa, and viruses), their genomes, and metabolic products [31]. The bacterial community (BC), that is, presence and abundance of such taxa, is influenced by diet composition, nutrient availability, and many environmental conditions. The microbes ferment parts of the diet, especially those parts that cannot be digested by the host's enzymes (allo‐ vs. auto‐enzymatic digestion). The fermentative end products are mostly short‐chained fatty acids (SCFA), which affect the intestinal epithelium, the host immune system and, via several pathways, the peripheral and central nervous system. This links the gastrointestinal microbiome and the host physiology in many ways. In mice, the BC in the cecum and colon is dominated on family level by *Muribaculaceae* and *Lachnospiraceae* [12, 32]. However, the former taxon is rarely described in humans and if so in low abundances. In humans, the families *Lachnospiraceae*, *Oscillospiraceae*, *Prevotellaceae*, and *Bacteroidaceae* have been described to be most abundant in fecal samples [33, 34]. It has to be noted that fecal samples do not fully represent the microbial community present in the intestinal sections, such that comparisons are limited.

As the gastrointestinal tract is the largest contact area of the immune system with the microbiome, local and systemic immune systems are primed here starting directly after birth [6]. Negative changes in the composition of the microbiome, often described, but badly defined as “dysbiosis,” contribute to pathophysiologic processes occurring in type‐2 diabetes, cardiac disease, obesity, and many other diseases [35]. Unsurprisingly, a specific and strongly different microbiome can lead to alterations in the outcome of animal experiments, consequently endangering reproducibility and standardization, as well as translation of results. In addition, the microbiome influences the host by modifying the metabolism and therefore toxicity of drugs [36, 37, 38]. This is one possible reason that contributes to a failing translation of promising preclinical results of drug development from animal models to humans [39].

While in humans and mice, the gastrointestinal microbiome is fairly well‐researched, only a few studies have been published on this topic in Syrian hamsters. Differences between mice and men, especially regarding their gastrointestinal anatomy, physiology, and microbiome, have been addressed, and these differences might possibly fail translation [1, 2, 3, 4, 5, 6]. Nevertheless, since Syrian hamsters are an important animal model in research, a better understanding of their microbiome is urgently needed. Consequently, further knowledge regarding the hamsters digestive physiology and a distinct analysis of differences between humans, mice, and hamsters is needed to potentially refine their diet recommendations.

This work provides insight into the microbiome throughout the complete gastrointestinal tract (i.e., content of nonglandular forestomach [FS], glandular stomach [GS], small intestinal [SIT], cecum [CAE], and colon [COL]) of 10 adult Syrian hamsters using 16S rRNA gene amplicon sequencing [24]. To understand the extent of similarity between two rodent species with a different gastrointestinal physiology, we compared the results obtained from hamsters to mice data generated previously [12], which have been analyzed with the same methods and pipeline from start to end in order to sustain comparability [40].

## Materials and methods

### Animals

Animals from a trial on amylase activity (ethics approval with reference no. 203‐25‐02‐20 from the Ethical Committee of the Veterinary Faculty, LMU Munich) [24] were used to obtain the samples for microbiome sequencing. The hamsters were housed following the relevant European and German animal welfare legislations (5.1‐2 31 5682/LMU/BMC/CAM 2019‐0007). They were kept under specific pathogen‐free (SPF) conditions in individually ventilated cages (Type IIIH; Tecniplast GmbH, Buggugiate, Italy) with aspen bedding (LASbedding PG3; Altromin Spezialfutter GmbH Co., Lage, Germany). Enrichment was available in the form of nesting material (5 × 5 cm; Nestlet, Datesand, UK), a red corner house (Tecniplast), and play tunnels (Datesand Ltd, Bredbury, Stockport, UK). The room climate was monitored (20–22 °C, relative humidity 45–55%, air exchange 11 times·h^−1^, HEPA‐filtered). The light cycle was constant with 12/12 h light/dark. Hygiene monitoring was performed every 3 months based on the recommendations of the FELASA‐14 working group.

Ten healthy female Syrian hamsters (outbred stock RjHan:AURA, 11 weeks old; breeder Janvier, France) were used. They were fed the standard diet used in the facility for at least 2 weeks before sacrifice (pelleted breeding diet for rats and mice). Table 1 gives the analyzed nutrient content of the batch used during the trial. Feed and sterilized water were available *ad libitum*. The hamsters were not fasted before sacrifice. They were euthanized by intraperitoneal injection of pentobarbital sodium at a dose of 500–800 mg·kg^−1^ body weight (Release 300 mg·mL^−1^; WDT, Garbsen, Germany).

**Table 1:** Analyzed nutrient composition of the pelleted diet fed to the hamsters.

Nutrient	Analyzed content, % (as‐fed basis)
Dry matter	90.9
Crude protein	21.8
Crude fat	4.7
Crude fiber	4.3
Crude ash	3.9
N‐free extracts	56.3

### Sampling of gastrointestinal content

Gastrointestinal samples were obtained from freshly sacrificed animals. To reduce contamination, the abdominal wall was disinfected with 80% ethanol before opening the body. Then, the linea alba was incised, and the skin and peritoneum were set aside and fixed with preparation needles. The gastrointestinal tract was pinched off at the esophagus 0.5 cm anterior of the stomach and at the end of colon in front of the pelvic bone. The gastrointestinal tract was then moved onto a sterile Petri dish (Sarstedt AG & Co. KG, Nümbrecht, Germany). Samples from the forestomach were obtained by opening the forestomach beginning at the esophagus following the small curvature and removing some ingesta using a sterile inoculating loop. The glandular stomach was sampled in similar fashion by opening the glandular stomach with a small incision at the large curvature and expanding the incision to around 0.5 cm. The ingesta was removed using a sterile inoculating loop. The ileum was sampled approximately 1 cm before the cecum. An incision was made and expanded to around 1 cm using small sterile scissors, and the ingesta was removed by gently moving the sterile inoculation loop over the intestinal surface. The cecum was incised and opened at the apex to around 0.5 cm to remove the digesta with a sterile inoculation loop. The colon was incised on the length of 1 cm behind the cecum and opened like the small intestine. Digesta had already formed fecal‐like pellets; thus, two to three pellets were removed using sterile forceps. All digesta samples were directly transferred into 600 μL Stool DNA Stabilizer (Invitek Molecular GmbH, Berlin, Germany) and stored at −20 °C until the DNA extraction was conducted.

### DNA extraction, library preparation, and sequencing of the 16S rRNA gene amplicons

Methodology for DNA extraction, library preparation, and 16S rRNA gene amplicon sequencing has been described in detail previously [12, 41]. In short, DNA was extracted using the modified protocol by Godon *et al*. [42]. Samples were thawed on ice, and microbial cell walls were lysed using both a chemical lysis step with 4 m guanidine‐thiocyanate and 5% *N*‐laurolylsarcosine and a mechanical lysis step conducted in Lysing Matrix Tubes B (MP Biomedicals, Eschwege, Germany) with the FastPrep 24™ (MP Biomedicals). Thereafter, DNA was cleaned up using the NuceloSpin gDNA cleanup kit according to the manufacturer's instructions (Macherey Nagel GmbH and Co. KG, Düren, Germany). Amplicon library preparation (V3–V4 region) and sequencing were described in detail previously [41]. Briefly, amplicons were purified using the AMPure XP system (Beckmann Coulter, Krefeld, Germany), and sequenced was conducted in a paired‐end mode (PE300; only using reads of 275 each) with pooled samples containing 10% (v/v) PhiX standard library in a MiSeq system (Illumina Inc., San Diego, CA, USA) prepared according to the manufacturer's instructions.

### Analysis of the 16S rRNA gene amplicons

Detailed analysis of 16S rRNA gene amplicon data has been described previously [43]. In short, fastq files were remultiplexed using a PERL script provided on the IMNGS website and analyzed using IMNGS [44]. Obtained operational taxonomic units (OTU) and zero‐radius operational taxonomic units (zOTU) were thereafter refined using silva [45] and mega x [46]. The provided taxonomy was rechecked using ezbiocloud [47], and taxonomy was further refined according to the International Nomenclature as provided on LPSN [48]. Downstream analyses were conducted in Rhea [49], a modular pipeline for microbial profiling of 16S rRNA gene amplicon sequencing data conducted in an r environment (version 4.3.1). The pipeline is available on the GitHub repository (https://github.com/Lagkouvardos/Rhea; accessed on February 12, 2023). The OTU and zOTU tables of all experimental samples can be found in Tables S1 and S2. The effective richness (i.e., effective number of species) and the effective Shannon diversity were calculated using OTUs [50], and further parameters were calculated using zOTUs. β‐diversity was calculated with generalized UniFrac distances [51]. *P* values were corrected for multiple testing using the Benjamini–Hochberg method [52], whereby values below 0.05 were considered statistically significant. For statistical testing, taxa and zOTUs with a prevalence equal to or more than 20% (proportion of samples positive for the respective taxa) in at least one of the groups and a relative abundance of equal to or more than 0.25% were considered. For multiple group testing, a Kruskal–Wallis rank sum test was conducted, and thereafter, a Wilcoxon rank sum test was conducted for pairwise comparisons. A nonlinear Fisher's exact test was used to determine the differences between samples with a low prevalence. Data were visualized using illustrator cs6 version 16.0.0 (Adobe Inc., San José, CA, USA). The most abundant taxa were visualized using the software prism version 2010 (GraphPad Software, San Diego, CA, USA).

## Results

### Description of the microbiome throughout the gastrointestinal tract of Syrian hamsters

The 16S rRNA gene amplification produced a total of about 1.6 × 10^6^ reads after OTUs abundance filtering with a mean of 33 648 (±19 952) reads per sample. After the removal of chimeras, *de novo* clustering based on plain sequence similarity (i.e., > 97%) provided a total of 321 OTUs. After denoising the amplicons, a total of 471 zero‐radius operational taxonomic units (zOTUs) were produced.

**Fig. 2:**
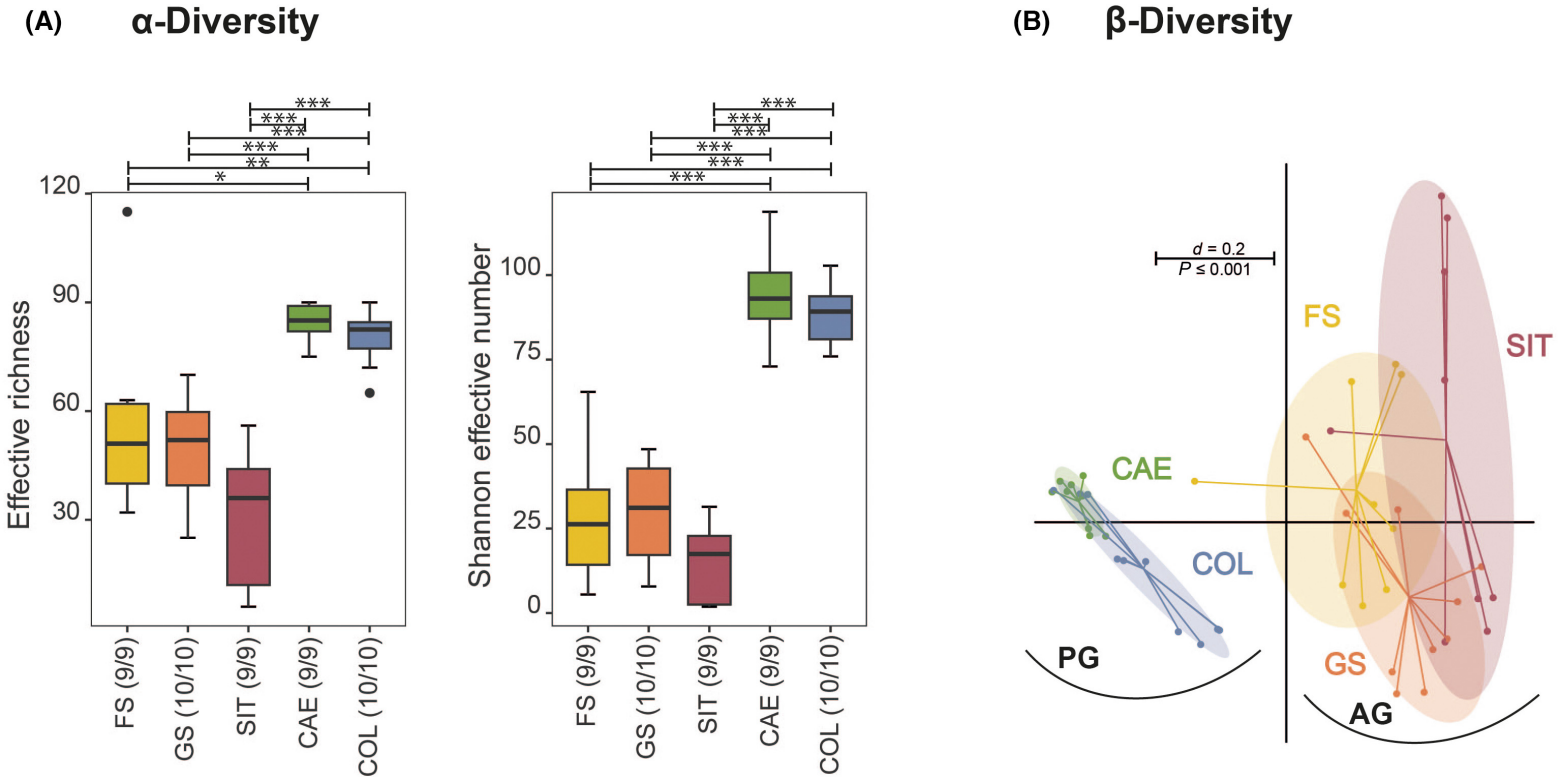
Comparison of α‐diversities and β‐diversities between different gastrointestinal regions; (A) α‐diversity (i.e., counted microbial diversity within a sample) shown as effective richness and Shannon effective numbers between samples from different gastrointestinal compartments; Boxplots depict the median (thick bar), upper and lower quartile (within the box), standard deviation (whiskers), and outliers (dots). Numbers in brackets behind the respective group indicate the number of individual mice. Significance is indicated by the lines and symbols above the graphs for pairwise comparison using the Wilcoxon rank sum test (*P ≤ 0.05; **P ≤ 0.01; ***P ≤ 0.001). (B) Multidimensional scaling plot showing the microbial β‐diversity (i.e., microbial diversity between different samples) for different gastrointestinal sites. The bar indicates percent difference, based on generalized UniFrac [51]. AG, anterior gastrointestinal compartments; CAE, cecum; COL, colon; FS, forestomach; GS, glandular stomach; PG, posterior gastrointestinal compartments; SIT, small intestinal tract.

Subsequently, the diversity of bacteria in a singular sample (i.e., α‐diversity) was assessed and compared between different gastrointestinal compartments using both the effective richness (i.e., richness normalized to 100 reads) and Shannon effective numbers (Fig. 2A). Of all compartments, SIT has the lowest species richness, followed by GS, FS, COL, and CAE in increasing order. When assessing the microbial profiles of the different gastrointestinal compartments, the samples obtained from ingesta originating from the anterior gastrointestinal compartments (AG; i.e., FS, GS, and SIT) were more similar than the samples obtained from ingesta originating from the posterior gastrointestinal compartments (PG; i.e., CAE and COL; Fig. 2B).

**Fig. 3:**
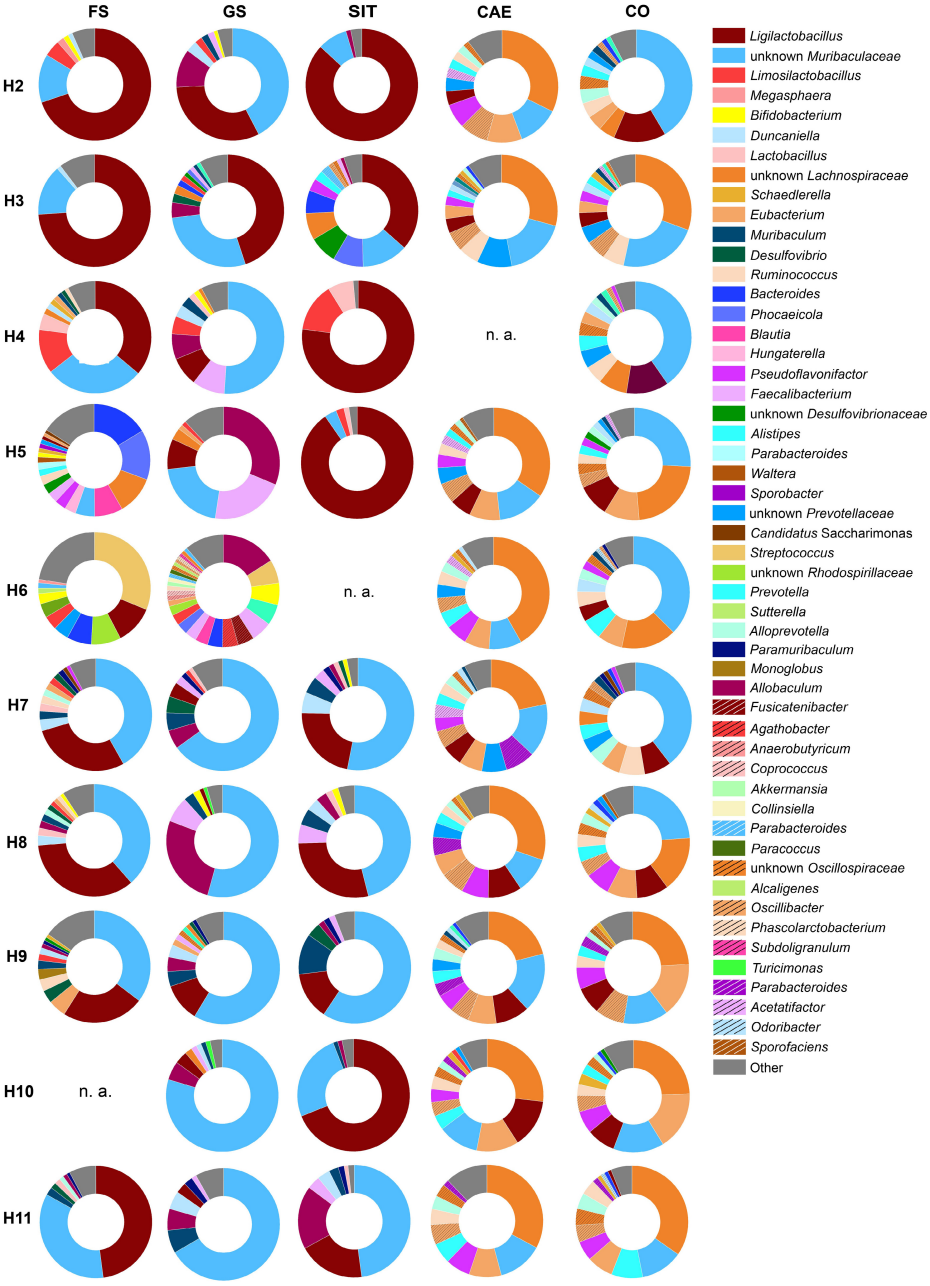
Composition of the bacterial community of 10 individual hamsters (H2–H11) on genus level for forestomach (FS), glandular stomach (GS), small intestine (SIT), cecum (CAE), and colon (COL). Locations where no sample was available due to not meeting the quality criteria were indicated as not available (n.a.).

To visualize the BC of individual animals, we plotted the composition of each sampling site per hamster at the genus level (Fig. 3). AG samples display either a higher relative abundance of Bacillota with the genera *Ligilactobacillus* and *Limosilactobacillus* or a higher relative abundance of an unknown member of the *Muribaculaceae*. Deviating samples are found in animals H5 and H6. Very obvious is the dominance of an unknown member of the *Lachnospiraceae* after the ingesta reached the PG, whereby some hamsters' COL BCs were dominated by an unknown member of the *Muribaculaceae* family (Fig. 3).

**Fig. 4:**
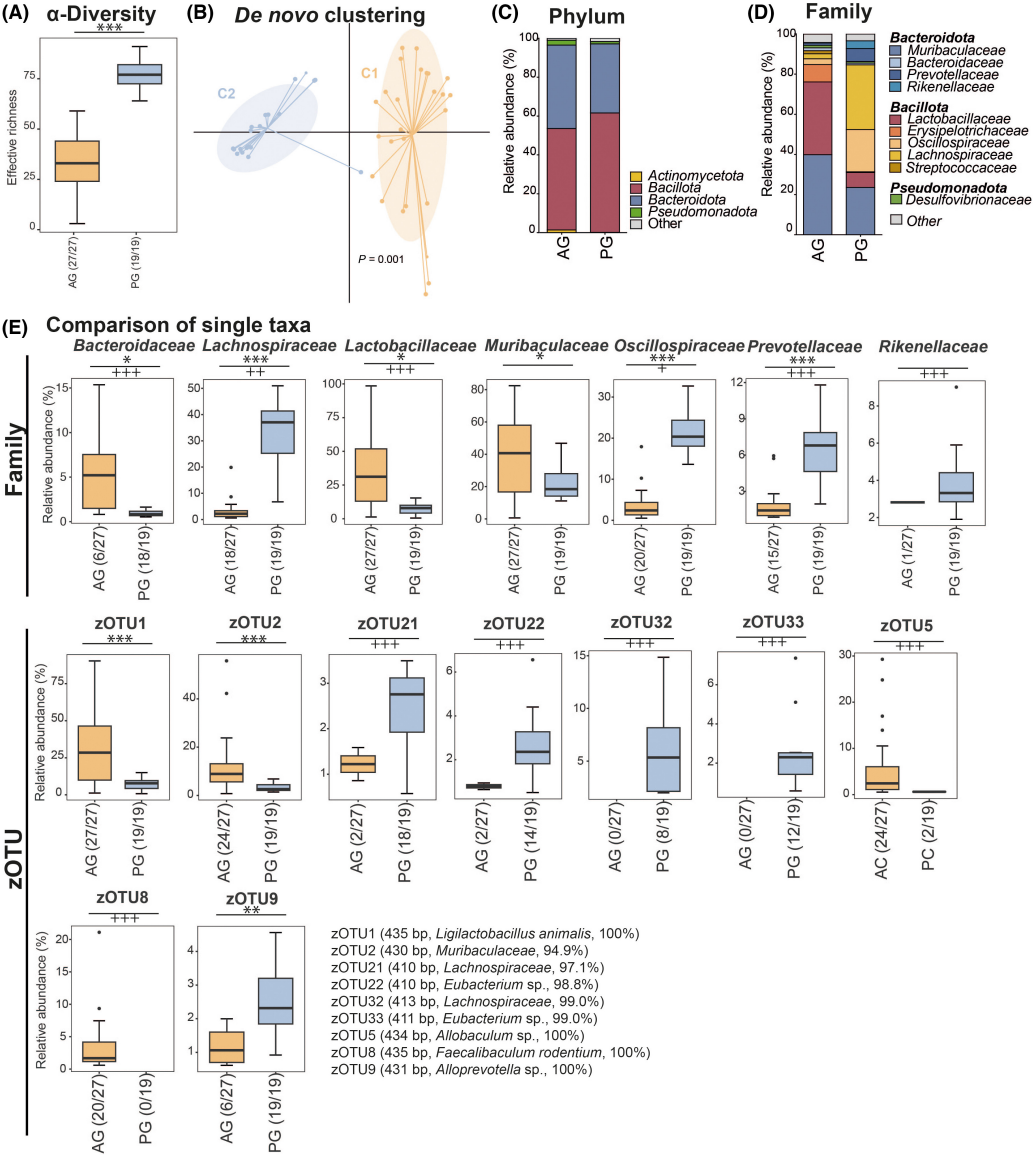
nterior and posterior gastrointestinal compartments (FS: *n* = 9; GS: *n* = 10, SIT: *n* = 9; CAE: *n* = 9, COL: *n* = 10); (A) α‐diversity displayed as effective richness between the gastrointestinal regions AG and PG; Boxplots depict the median (thick bar), upper and lower quartile (within the box), standard deviation (whiskers), and outliers (dots); (B) *de novo* clustering of the microbial profiles obtained from samples of all gastrointestinal regions using two clusters according to the highest Calinski–Harabasz (CH) index; significance was tested using PERMANOVA; (C) Phylum level composition of AG and PG samples (mean); (D) Family‐level composition of AG and PC samples (mean); (E) Comparison of single taxa differing significantly between AG and PG; zOTUs (zero‐radius operational taxonomic units) were displayed only when their relative abundance was above 2% for one group; zOTUs were identified by ezbiocloud [47]; the text to the right shows sequence length, the closest taxon matching, and sequence similarity; Boxplots indicate median (thick bar), upper and lower quartile (within box), and standard deviation (whiskers). Dots are outliers. Numbers in brackets behind the respective group indicate the number of individual samples. Significance is indicated by the lines and symbols above the graphs for pairwise comparison (stars, Wilcoxon‐Rank‐Sum test; crosses, Fisher's Exact test; */+ *P* ≤ 0.05; **/++ *P* ≤ 0.01; ***/+++ *P* ≤ 0.001). AG, anterior gastrointestinal compartments; PG, posterior gastrointestinal compartments.

In general, the AG seemed to display a lower bacterial richness than the PG (Fig. 4B). Further BC of the AG was composed of Bacillota and Bacteroidota, while the PG was slightly more dominated by the phylum Bacillota. Other phyla contributed to the composition of the BC only in small numbers (Fig. 4C). At the family level, the differences between AG and PG became more distinct. In the AG, the families Muribaculaceae, Lactobacillaceae, and Erysipelotrichaceae contributed to about 80% of BC, while in PG, the strongest fraction was Lachnospiraceae, followed by Muribaculaceae, Oscillospiraceae, and Lactobacillaceae (Fig. 4D). The higher number of the latter in AG could be traced to some molecular species. For instance, different molecular species originating from the genus *Lactobacillus* (i.e., zOTU1, zOTU10, and zOTU14) displayed higher relative abundances (Fig. 4E). Interestingly, both gastrointestinal compartments displayed a high abundance of Muribaculaceae, but they differed on molecular strain‐level composition. Nevertheless, the higher abundance of the family Lachnospiraceae in the PG was due to matching zOTUs, which were significantly higher in PG than in AG (i.e., zOTU16, zOTU20, zOTU21, zOTU32, zOTU44, and zOTU61).

**Fig. 5:**
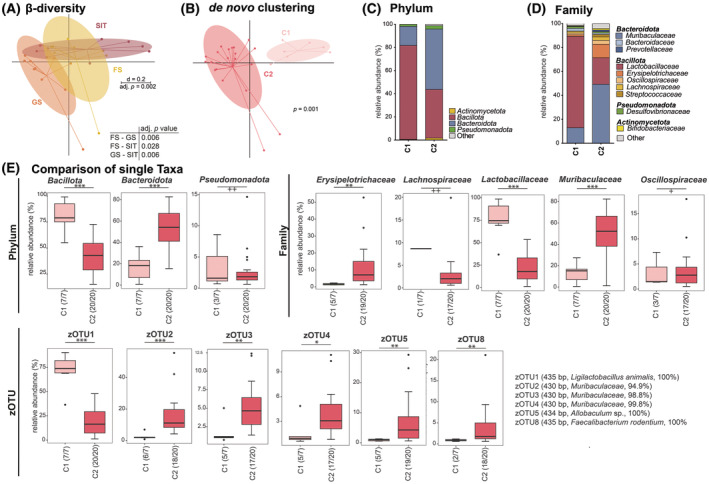
Comparison of the bacterial communities between AG and the clusters defined for AG (FS: *n* = 9; GS: *n* = 10, SIT: *n* = 9); (A) multidimensional scaling (MDS) plot showing the β‐diversity for the three AG sampling sites based on generalized UniFrac [51]; The scale indicates a 20% difference (*d* = 0.2); significance was tested using PERMANOVA; (B) *de novo* clustering of the microbial profiles obtained from samples of all gastrointestinal regions using two clusters according to the highest Calinski–Harabasz (CH) index for all AG samples into two clusters, Cluster AG‐1 (C1) and Cluster AG‐2 (C2); significance was tested using PERMANOVA; (C) Bacterial communities in AG Cluster AG‐1 and AG‐2, on phylum level, displayed as mean; (D) As in panel C, on family level; (E) Comparison of single taxa differing significantly between Cluster AG‐1 and AG‐2 samples for different taxonomic levels; zOTUs (zero‐radius operational taxonomic units) were displayed only when their relative abundance was above 2% for one group; zOTUs were identified by ezbiocloud [47]; the text to the right shows sequence length, the closest taxon matching, and sequence similarity; Boxplots indicate median (thick bar), upper and lower quartile (within box), and standard deviation (whiskers). Dots are outliers. Numbers in brackets behind the respective group indicate the number of individual samples. Significance is indicated by the lines and symbols above the graphs for pairwise comparison (stars, Wilcoxon rank sum test; crosses, Fisher's exact test; */+ *P* ≤ 0.05; **/++ *P* ≤ 0.01; *** *P* ≤ 0.001). AG, anterior gastrointestinal compartments; FS, forestomach; GS, glandular stomach; SIT, small intestinal tract.

In general, in AG, the FS seemed to display the highest effective richness, followed by the GS and the SIT (Fig. 2A). The β‐diversity displayed significant differences between all AG, namely FS, GS, and SIT (Fig. 5A). Nevertheless, clustering observed in the multidimensional scaling (MDS) plot in Fig. 5A compared with *de novo* clustering (Fig. 4B) indicated that the assignment based on sampled locations (i.e., FS, GS, and SIT) does not always correlate with composition. Indeed, the highest CH‐Index was found for two clusters only, even though three compartments were sampled. *De novo* clustering using two clusters identified the samples H2 FS and H2 SIT, H3 FS and H3 SIT, H4 SIT, H5 SIT, and H10 SIT to form Cluster AG‐1, while all other samples were found in Cluster AG‐2. Cluster AG‐1 was strongly dominated by the phylum Bacillota (81.2%, Fig. 5C) comprised mostly of the family Lactobacillaceae (Fig. 5D). In comparison, Cluster AG‐2 displayed a lower relative abundance of Bacillota (42.3%, Fig. 5C), but three times the relative abundance of Bacteroidota (52.2%, Fig. 5C). This difference was evident even at molecular strain level where Cluster AG‐1 contained 75% of zOTU1 (*Ligilactobacillus animalis*, 100%). Other molecular strains displaying significant differences were found in higher relative abundances in Cluster AG‐2 or only in this cluster (Fig. 5E). Furthermore, phylum Bacillota in Cluster AG‐2 displayed a higher diversity on family level compared to Cluster AG‐1 and included the families Lactobacillaceae, Erysipelotrichaceae, Oscillospiraceae, and Lachnospiraceae in descending abundance (Fig. 5D).

**Fig. 6:**
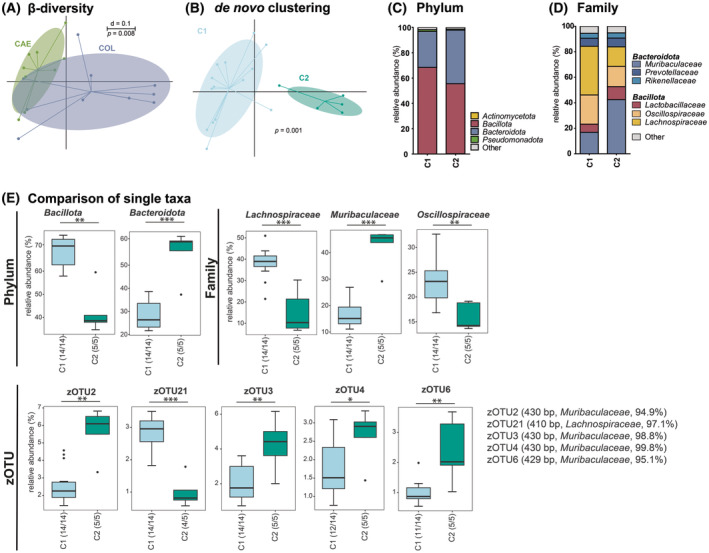
Comparison the bacterial communities between PG and the clusters defined for PG (CAE: *n* = 9, COL: *n* = 10) (A) multidimensional scaling (MDS) plot showing the β‐diversity for the three PG sampling sites based on generalized UniFrac [51]. The scale indicates a 20% difference (*d* = 0.2); significance was tested using PERMANOVA; (B) *de novo* clustering of the microbial profiles obtained from samples of all gastrointestinal regions using two clusters according to the highest Calinski–Harabasz (CH) index for all PG samples into two clusters, Cluster PG‐1 (C1) and Cluster PG‐2 (C2); significance was tested using PERMANOVA; (C) Bacterial communities in PG Cluster PG‐1 and PG‐2, on phylum level, displayed as mean; (D) As in panel C, on family level; (E) Comparison of single taxa differing significantly between Cluster PG‐1 and PG‐2 samples for different taxonomic levels; zOTUs (zero‐radius operational taxonomic units) were displayed only when their relative abundance was above 2% for one group; zOTUs were identified by ezbiocloud [47]; the text to the right shows sequence length, the closest taxon matching, and sequence similarity; Boxplots indicate median (thick bar), upper and lower quartile (within box), and standard deviation (whiskers). Dots are outliers. Numbers in brackets behind the respective group indicate the number of individual samples. Significance is indicated by the lines and symbols above the graphs for pairwise comparison (stars, Wilcoxon‐Rank‐Sum test; crosses, Fisher's Exact test; *P ≤ 0.05; **P ≤ 0.01; ***P ≤ 0.001). CAE, cecum; COL, colon; PG, posterior gastrointestinal compartments.

The PG was also checked for differences in either the sampling site or using *de novo‐*generated clusters. Significant differences were found for CAE and COL in their microbial diversity (Fig. 6A). Nevertheless, we again used *de novo* clustering to gain further insight (Fig. 6B). Cluster PG‐2 was characterized by a higher number of the phylum Bacteroidota and the family Muribaculaceae, respectively, compared with Cluster PG‐1 (Fig. 6C,D). We found a higher relative abundance of species belonging to the family Muribaculaceae and also a higher diversity within this taxon in Cluster PG‐2 (Fig. 6E).

In summary, we observed strong differences in the BC of the anterior (AG, i.e., FS, GS, and SIT) and the posterior gastrointestinal parts (PG, i.e., CAE and COL). At phylum level, the relative abundances of the Bacteroidota were higher in AG, while Bacillota was higher in PG.

### Comparison of the gastrointestinal bacterial community between Syrian hamsters and C57BL/6J mice

As previously mentioned, an important point in laboratory animal science is to identify which species are used best for specific experiments. Mice and hamsters show some similarities in their digestive physiology and are even fed similar or even the same cereal‐based diets in laboratory animal facilities without clinical disturbances. Thus, we aimed to compare the microbiome of hamsters to mice from previous work [12]. To limit the influence of diet as a confounder, we chose mice fed a pelleted diet most similar to that of the hamsters in this study [12, 53].

**Fig. 7:**
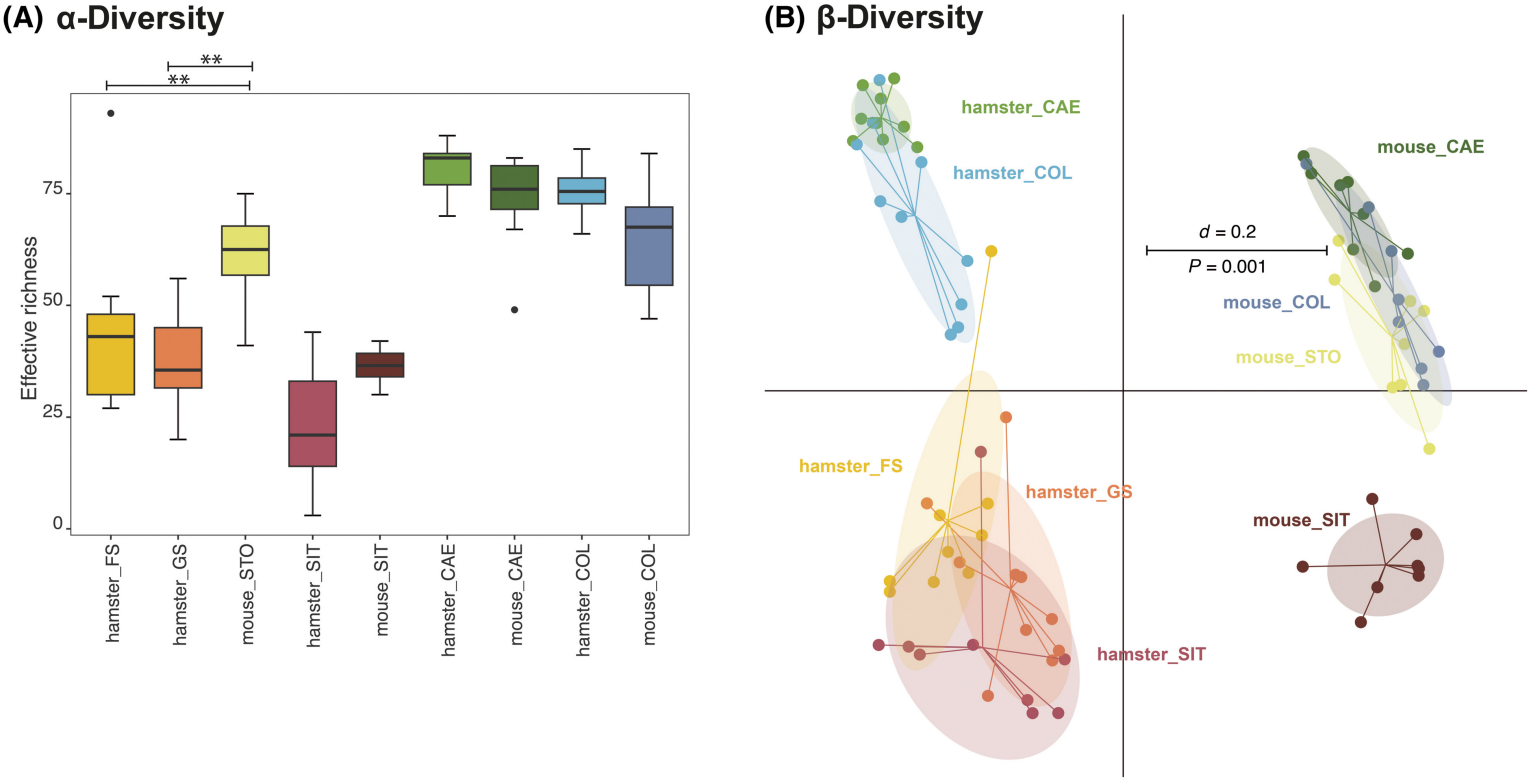
Comparison of hamsters and mice bacterial communities for α‐diversity β‐diversity. (A) Comparison of hamsters and mice for α‐diversity shown as effective richness from different gastrointestinal compartments. Boxplots depict the median (thick bar), upper and lower quartile (within the box), standard deviation (whiskers), and outliers (dots); Significance is indicated by the lines and symbols above the graphs (Mann–Whitney‐U Test), **P ≤ 0.01; (B) multidimensional scaling (MDS) plot showing the β‐diversity for hamster and mouse samples. The scale indicates a 20% difference in composition (*d* = 0.2); significance was tested using PERMANOVA. CAE, cecum; COL, colon; FS, forestomach; GS, glandular stomach; SIT, small intestinal tract; STO, mouse stomach.

Mice and hamsters display an overall comparable bacterial richness in the different gastrointestinal compartments. However, the two species possess strongly different microbial profiles (Fig. 7).

**Fig. 8:**
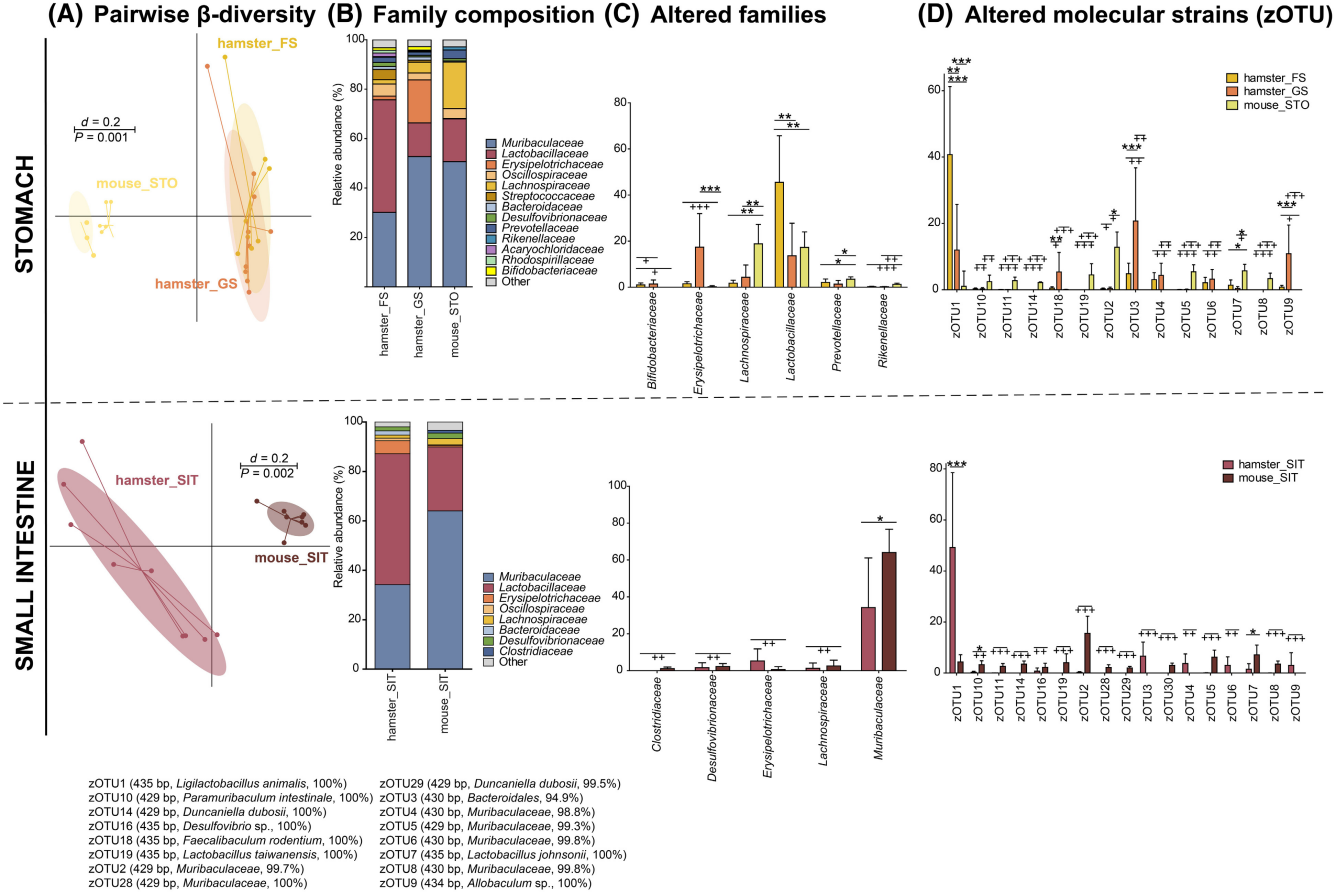
Differences in microbiological composition for samples taken from hamsters and mice. (A) Stomach samples, comparing forestomach and glandular stomach versus mouse stomach and (B) small intestinal tract samples from hamsters and mice, respectively. From left to right, multidimensional scaling (MDS) plots displaying the β‐diversity based on generalized UniFrac distances (scale indicates differences between samples; i.e., *d* = 0.2 is 2% difference) [51], significance was tested using PERMANOVA; the composition of the bacterial communities in AC samples on the family level (mean values), altered families, and altered molecular strains (zero‐radius operational taxonomic units, zOTU); bar plots indicate mean, whiskers indicate standard deviation; (C) zOTUs were identified by ezbiocloud [47]; the text to the right shows sequence length, the closest taxon, and the sequence similarity of the zOTUs. Significance is indicated by the lines and symbols above the graphs for pairwise comparison (stars, Wilcoxon‐Rank‐Sum test; crosses, Fisher's Exact test; */+ *P* ≤ 0.05; **/++ *P* ≤ 0.01; ***/+++ *P* ≤ 0.001). FS, forestomach; GS, glandular stomach; SIT, small intestinal tract; STO, mouse stomach.

Next, we compared each gastrointestinal compartment. The murine stomach (STO) was dominated by the families Muribaculaceae, Lachnospiraceae, and Lactobacillaceae, while in the hamsters' GS, the families Muribaculaceae, Erysipelotrichaceae, and Lactobacillaceae occurred most frequent. In contrast, the hamsters' FS was dominated by the family Lactobacillaceae followed by Muribaculaceae. Concerning the molecular strain level, the difference between the mouse STO and the hamsters' GS seemed to be less strong than the difference between the mouse STO and the hamsters' FS. Nevertheless, various zOTUs are present in only one of the two rodent species (Fig. 8A).

Similarly, the microbial profiles of the SIT displayed significant differences in their microbial profiles and composition. The murine SIT was dominated by the family Muribaculaceae followed by Lactobacillaceae. In contrast, the hamsters' SIT was in large parts composed of Lactobacillaceae followed by Muribaculaceae and a small fraction of Erysipelotrichaceae. While the comparison of relative abundance for the family Lactobacillaceae was not significant between hamsters and mice SIT, Muribaculaceae and Erysipelotrichaceae displayed significant differences. Interestingly, at the family level, no significant difference for Lactobacillaceae was found, and significant differences for molecular strains (i.e., zOTUs) within this family were observed. Here, in hamsters' SIT, zOTU1 was dominant, and only small numbers of other zOTUs belonging to this family (i.e., zOTU3) were observed. In contrast, mice had a wider variety of molecular strains of the Lactobacillaceae. Regarding the molecular strains of the Muribaculaceae, the strains showed larger species specificity, that is, being more common in hamsters or mice (Fig. 8B).

**Fig. 9:**
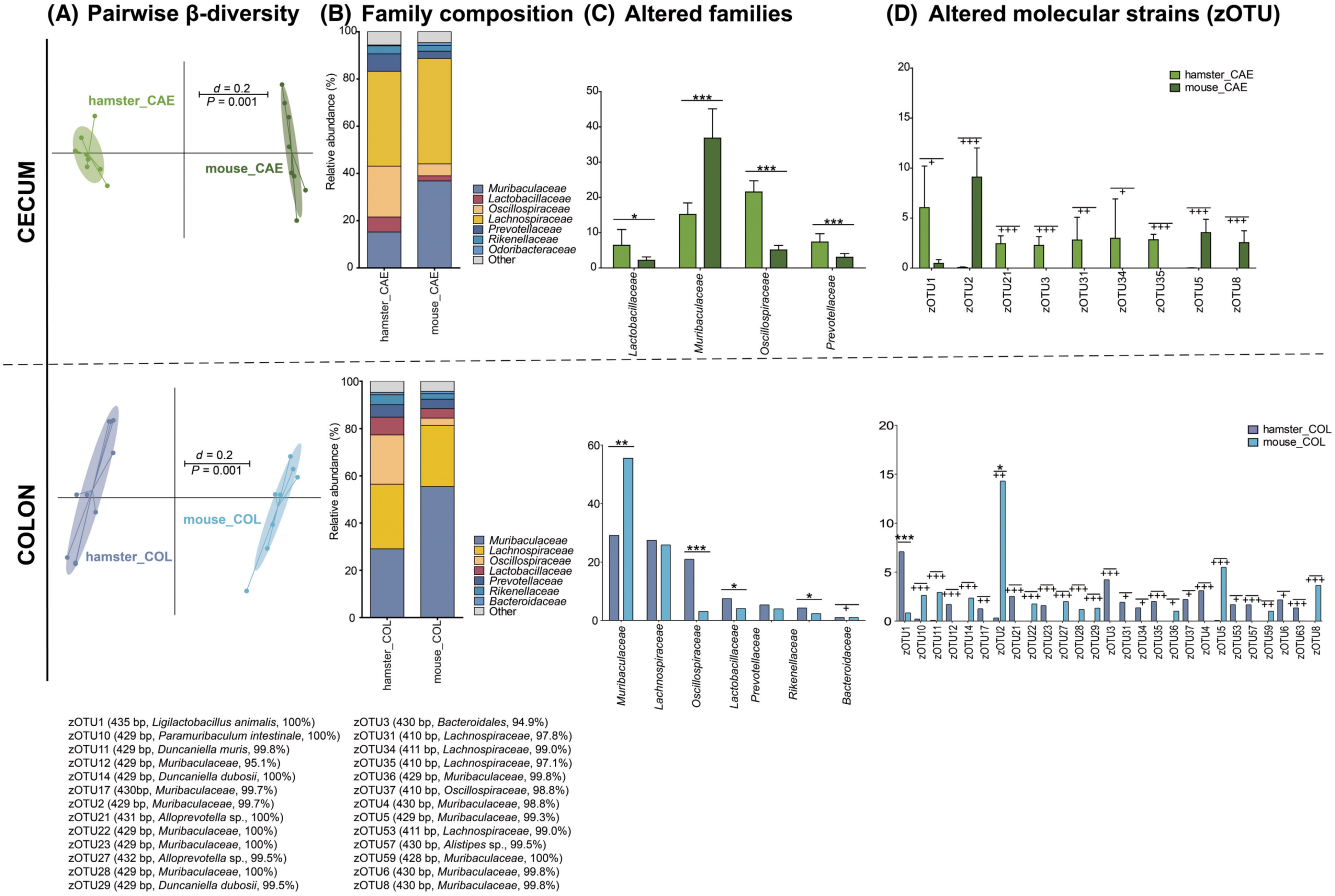
Differences in microbiological composition for samples taken from hamsters and mice. (A) Cecum samples, comparing hamsters and mice, and (B) Colon samples from hamsters and mice, respectively. From left to right, multidimensional scaling (MDS) plots displaying the β‐diversity based on generalized UniFrac distances (scale indicates differences between samples; i.e. *d* = 0.2 is 20% difference) [51], significance was tested using PERMANOVA; the composition of the bacterial communities in PG samples at the family level (mean values), altered families, and altered molecular strains (zero‐radius operational taxonomic units, zOTU); bar plots indicate mean, whiskers indicate standard deviation; (C) zOTUs were identified by ezbiocloud [47]; the text to the right shows sequence length, the closest taxon, and the sequence similarity of the zOTUs. Significance is indicated above for pairwise comparison (stars, Wilcoxon rank sum test; crosses, Fisher's exact test; */+ *P* ≤ 0.05; **/++ *P* ≤ 0.01; ***/+++ *P* ≤ 0.001). CAE, cecum; COL, colon.

In both PG, that is, cecum and colon, the microbial profiles of the two rodent species differed significantly. In both CAE and COL, the hamsters' BC was dominated by Lachnospiraceae and Oscillospiraceae. Muribaculaceae displayed a higher relative abundance in the BC of COL than in the BC of CAE (Fig. 9A,B). In mice, the BC of CAE was dominated by Lachnospiraceae and Muribaculaceae, while Oscillospiraceae were observed in low abundances only (Fig. 9A). The BC of COL was mostly composed of Muribaculaceae followed by Lachnospiraceae (Fig. 9B). As observed in the AG, prominent differences were observed due to the presence and absence of specific zOTUs in the two rodent species (Fig. 9A,B). For all compartments, hamsters showed a higher number of species than mice (Fig. 7A).

To summarize, hamsters and mice showed distinctly different microbial profiles in all gastrointestinal compartments. Differences are mainly related to bacteria in the families Muribaculaceae, Lactobacillaceae, and Lachnospiraceae. In all locations, mice displayed stronger relative abundances of Muribaculaceae, while hamsters displayed stronger relative abundances of Lactobacillaceae in the FS and SIT. In the hindgut, relative abundances of Lachnospiraceae were somewhat comparable between mice and hamsters, whereby mice showed a stronger relative abundance of Muribaculaceae and hamsters of Lachnospiraceae and Oscillospiraceae.

## Discussion

The microbiome not only influences the host's physiology and the manifestation of pathologies, but the distinct knowledge of the microbiome in rodent models is necessary to choose a suitable animal model. Recent work described the microbiota influencing the metabolism of drugs and consequently their impact on the host [36, 37, 38]. This is even more important since the microbiome of rodents and humans is hardly comparable and the translation of results from rodent models might consequently fail [39]. Hamsters have become an increasingly important model organism recently. Currently, the bacterial community in various compartments of the gastrointestinal tract in Syrian hamsters has not been described, while the mouse microbiome has been studied in some detail.

In the forestomach of the hamsters, no secretory activity of enzymes takes place, and various mechanical, chemical, and biological functions have been suggested, which are reviewed in detail elsewhere [54]. It seems plausible that dietary starch is degraded to a small extent by salivary amylase and to a higher extent by microbial fermentation in the forestomach [22]. The comparatively high pH level in the forestomach would allow metabolically active bacteria to ferment substrate in the forestomach [23, 24], while the pH in the mouse stomach is low. Indeed, Lactobacillaceae and Muribaculaceae were found to be the dominating species (Fig. 8A), which have been associated with the capacity for starch digestion [9, 55]. Besides, species from the Lactobacillaceae in the forestomach might provide protection, for example, having antifungal properties and suppressing enteropathogenic microbes, especially in this food‐hoarding species [56, 57, 58].

In the glandular stomach of hamsters, the pH is low (approx. 2.0), and only few microorganisms can survive in this milieu. Despite this hostile environment, the effective richness of the bacterial community did not differ significantly between the forestomach and the glandular stomach content samples in this study. One could assume that we detected only the genetic material and not living bacteria using 16S rRNA sequencing, but the bacterial composition observed was significantly different between FS and GS, which supports the functional difference of these two stomach compartments and, for example, the presence of specific and alive bacteria (Fig. 8A).

The next gastrointestinal compartment is the small intestine, that is, the ileum. In the hamsters shown here and in mice [12, 32], the SIT had a relatively low bacterial richness. The major dietary source of energy, starch, is digested by pancreatic amylase in the duodenum, then absorbed in the form of glucose and oligosaccharides. In hamsters, the pancreatic amylase activity is significantly lower than in rats and mice [24]. However, as a granivorous species, the hamster tolerates high starch diets, and no clinical digestive problems on common cereal‐based laboratory diets have been reported. The beginning enzymatic digestion and microbial fermentation in the forestomach, as mentioned above, might play a role by ensuring the inflow of better degradable starch into the small intestine. The effectiveness of salivary amylase and the forestomach fermentation needs to be clarified in further studies. Nevertheless, Lactobacillaceae in the ileum observed in this work might still contribute to the digestion of starches throughout the small intestinal tract (Fig. 8B).

The different digestive functions of the three prececal sections in hamsters (i.e., FS, GS, and SIT) resulted in specific BCs (Fig. 4B). However, when using *de novo* clustering, samples were divided according to their phylogeny in either a cluster with samples dominated by Lactobacillaceae (Cluster 1 AC) or Muribaculaceae (Cluster 2 AC). For mice, a high similarity in the microbiome of gastrointestinal regions before the cecum has previously been described [32]. Both findings underline that fecal samples hardly allow conclusions regarding the anterior parts of the gastrointestinal tract. Since specific fermentative processes and metabolic end‐products dominate each gastrointestinal region, the use of fecal samples for research purposes gives only very limited insight into the microbiome–host interactions.

Hamsters are hindgut fermenters with an enlarged cecum that serves as the major site for microbial fermentation of carbohydrates and fiber [20], while it can also retain ingesta [21]. The bacterial composition in the CAE is in accordance with this (Fig. 5C). Indeed, Lachnospiraceae and Oscillospiraceae detected here are associated with the fermentation of resistant starch and fiber [59, 60, 61]. This concurs with high amounts of starch found in the cecum content of hamsters, similar to that of mice and rats [20, 24].

In the colon content of hamsters, few starch particles were found, which indicates that the major part of the dietary starch has already been fermented and digested in the anterior gastrointestinal compartments [24]. Nevertheless, microbiota from the cecum will probably spill into the colon, contributing to the genetic material of the bacterial community we observed here (Fig. 2). Whether these microbiota still can provide any function in further fermenting fibers in the colon must remain open.

The mean retention time of ingesta in the gastrointestinal tract of hamsters is shorter than that in rabbits, rats, and guinea pigs. However, the total tract diet digestibility in hamsters is quite high, even higher than in rabbits and rats [62]. This may suggest a high overall efficiency of both auto‐enzymatic digestion and microbial fermentation in this species.

As the microbiota in rodent models not only influences the host's physiology and the manifestation of pathologies, the distinct knowledge of the microbiota in rodent models is necessary to understand these processes and choose the most suitable animal model. This will consequently allow the correct evaluation and interpretation of results obtained from animal experiments. Additionally, recent work described the microbiota influencing the metabolism of drugs and their impact on the host [36, 37, 38]. This fact has to be taken into account as the microbiome of rodents and humans is hardly comparable and the translation of results from rodent models might consequently fail, as previously described [39].

On account of the comparability of rodent models, we compared the results of the hamsters to previous data on mice. Data obtained from a previous trial conducted by the authors using mice with no further intervention were used [12]. Although the factor of housing may influence the gut microbiome [11], it can hardly be overcome as experiments are conducted globally in different animal facilities. The β‐diversity displayed vastly different microbial profiles between hamsters and mice sites (Fig. 7B). More importantly, while in hamsters, we observed a separation of BC profiles before and after the cecum, in mice such a separation was not observed. In mice, we found the STO, CAE, and COL to cluster together, while the SIT clustered apart. Contrasting results have been reported by Lkhagva *et al*. [32], that is, the microbiome of C57BL/6J mice before and after the cecum differed significantly. These contrasting results might be explainable by increased coprophagy in a mouse cohort. In contrast, hamsters do not usually show coprophagy when fed highly digestible laboratory diets, which concurs with our results of the AG microbiome differing markedly from the hindgut compartments. We also assume that the mice in our experiment conducted more coprophagy, but this was neither specifically monitored nor prohibited in the trial. Taken together, almost all differences observed between the two rodent species were to be expected when considering their differences in gastrointestinal anatomy and digestive physiology.

Of note, the characterization of bacterial members of the microbiome using more in‐depth analyses such as other omics or culturing approaches is needed to further understand the functional contribution of the microbiota to the digestive physiology in hamsters. We found, for instance, that many molecular strains of the Muribaculaceae in this study seemingly have not been classified on genus level and were different for hamsters and mice, but this bacterial family is important in laboratory rodents [8, 9, 63, 64], which will finally influence the translation into humans.

## Conclusion

This work gives a first insight into the composition of the bacterial community of healthy hamsters throughout the gastrointestinal tract using 16S rRNA gene amplicon data. The observations suggest that the forestomach of hamsters is a first microbial fermentation chamber where starch is most likely fermented by members of the families Lactobacillaceae and Muribaculaceae. Furthermore, the data provide a reference dataset for other experiments and aid in a better understanding of hamsters' digestive physiology. Consequently, the data contribute to a better characterization of this laboratory animal and allows better experimental planning. By comparing the bacterial community of the gastrointestinal tract of hamsters with that of mice, a better understanding of differences in the bacterial community and digestive functions between these two rodent models is possible, contributing to a better translation from animal models to humans. Further studies are warranted to investigate the bacterial communities in common laboratory animal species at a functional level [8, 65].

## Conflict of interest

The authors declare no conflict of interest.

## Author contributions

LFB and JW were involved in conceptualization and investigation. LFB, DM, and JW were involved in methodology. JW was involved in formal analysis, data curation, writing—original draft preparation, and visualization. LFB and BP were involved in resources and project administration. LFB, BP, and KN were involved in writing—review and editing. All authors have read and agreed to the published version of the manuscript.

## Supporting information


**Table S1.** OTUs observed in the respective gastrointestinal regions of hamsters.

**Table S2.** zOTUs observed in the respective gastrointestinal regions of hamsters.

## Data Availability

The 16S rRNA gene amplicon data generated from the gastrointestinal content of Syrian hamsters have been archived on the Sequence read archive under the accession code PRJNA1056556 and 16S rRNA gene amplicon data generated from the gastrointestinal content from C57BL/6J mice under the accession code PRJNA700529.
